# The Role of Lung Ultrasound in the Management of the Critically Ill Neonate—A Narrative Review and Practical Guide

**DOI:** 10.3390/children8080628

**Published:** 2021-07-24

**Authors:** Lukas Aichhorn, Erik Küng, Lisa Habrina, Tobias Werther, Angelika Berger, Berndt Urlesberger, Bernhard Schwaberger

**Affiliations:** 1 Comprehensive Center for Paediatrics, Department of Paediatrics and Adolescent Medicine, Division of Neonatology, Paediatric Intensive Care & Neuropaediatrics, Medical University of Vienna, Währinger Gürtel 18-20, 1090 Vienna, Austria; erik.kueng@meduniwien.ac.at (E.K.); lisa.habrina@meduniwien.ac.at (L.H.); tobias.werther@meduniwien.ac.at (T.W.); angelika.berger@meduniwien.ac.at (A.B.); 2 Department of Paediatrics and Adolescent Medicine, Division of Neonatology, Medical University of Graz, Auenbruggerplatz 34/2, 8036 Graz, Austria; berndt.urlesberger@medunigraz.at (B.U.); bernhard.schwaberger@medunigraz.at (B.S.)

**Keywords:** lung ultrasound, neonatal resuscitation, transition process, respiratory distress syndrome

## Abstract

Lung ultrasound makes use of artifacts generated by the ratio of air and fluid in the lung. Recently, an enormous increase of research regarding lung ultrasound emerged, especially in intensive care units. The use of lung ultrasound on the neonatal intensive care unit enables the clinician to gain knowledge about the respiratory condition of the patients, make quick decisions, and reduces exposure to ionizing radiation. In this narrative review, the possibilities of lung ultrasound for the stabilization and resuscitation of the neonate using the ABCDE algorithm will be discussed.

## 1. Introduction

On a daily basis, neonatologists are confronted with the challenging task of achieving the optimal care for term and preterm neonates. In particular, the stressful situation of resuscitation of infants often requires quick and decisive action with potentially crucial decisions regarding the current condition, but also the long-term outcome of the patient, based on limited information. The majority of these emergency situations in neonatology are respiratory conditions, often during the process of neonatal transition [1,2].

In the last decade, the use of lung ultrasound (LUS) has gained momentum in various specialties, not only to reduce exposure to radiation but because of the broad spectrum of diagnostic possibilities of this formerly underappreciated technique, especially in neonatal intensive care units (NICU) [3].

Many aspects of the management of critically ill neonates are controversial. As a consequence, a large number of protocols for the postnatal stabilization of infants have been proposed, to the extent that almost every institution has developed its own guideline and standard of practice for this purpose. In adult patients, ultrasound has become an integral part of emergency medicine [4] and pre-hospital care [5] over the past two decades with the corresponding standardization of protocols. However, only with recent publication of the International Evidence-Based Guidelines on Point of Care Ultrasound by the European Society of Paediatric and Neonatal Intensive Care, ultrasound found its way into paediatric and neonatal intensive care units in a standardized form [6]. The Paediatric Life Support (PLS) guidelines by the European resuscitation council mention ultrasound for situations like confirmation of proper tracheal tube position, management of circulatory failure or diagnosis of tension pneumothorax, pneumonia and pericardial tamponade. However, the European Resuscitation Council Guidelines 2021 for Newborn resuscitation and support of transition of infants at birth (NLS Guidelines) do not consider ultrasound, mainly due to a lack of evidence for term and preterm infants [7]. Several methods for opening and securing the airway mentioned in the NLS Guidelines can be assessed using ultrasound.

In our units, we started using a standardized LUS protocol, starting first in Vienna in 2016 [8], since LUS has been proven to be an effective, reliable, cheap—and frankly—a reasonably easy way to gain knowledge about the respiratory condition of a patient [3,9,10].

In this narrative review we want to share our insights, experiences and evidence-based information about the benefits of LUS in the management of the critically ill neonate. We will provide a guide through the stabilization process using the ABCDE algorithm. The PLS guidelines use A, B, C, D and E as mnemonic to assess the critically ill child and neonate, where A stands for airway, B for breathing, C for circulation, D for disability and E for other emergencies or environment [7]. 

Lung ultrasound makes use of *artifacts* generated by the ratio of air and fluid in the lung. For a long time, maybe even because of this particular feature, LUS has not been in the focus of research. One of the first reports of using LUS was published in 1951 by Stuhlfauth et al., who reported typical reflections in cases of pneumothorax. Since then, several studies were published with limited impact [11,12,13]. After gaining momentum in adult critical care medicine in the early 21st century, “International evidence-based recommendations for point of care lung ultrasound“ were published in 2012 [6]. The paper provided the reader with evidence-based recommendations regarding the application of LUS in case of pneumothorax, interstitial syndrome, lung consolidation and pleural effusion. In the following years and especially recently, triggered by the SARS-CoV-2 pandemic, an enormous increase of research regarding LUS has emerged.

In this review, we focus on point of care LUS examinations performed by neonatologists in the intensive care setting, therefore we chose the term neonatologist-performed-LUS (NPLUS) in analogy to the common term of neonatologist-performed echocardiography (NPE).

The detailed description of the signs used in LUS for the neonate as well as certain applications of NPLUS have been sufficiently described elsewhere and are beyond the scope of this review [3,8,9,14,15]. However, for better understanding we briefly want to address the most important signs:

The first step in lung ultrasound is looking for lung sliding, which represents the movement of parietal and visceral pleura. In a healthy lung, a thin, regular pleural line is seen, with horizontal, hyperechogenic reverberation artefacts underneath, known as A-Lines. In case of a shift from a healthy lung to a less aerated lung, usually B-Lines emerge. Those can be identified as a well-defined comet-tail artefact, which erases A-Lines and moves with lung sliding. Newborns present initially with B-Lines, with a declining number of B-Lines in case of a normal transition during the first hours of life. In case of a very poorly or not aerated lung area, consolidations may be observed. In this case, hypoechoic areas, or even hepatization of the lung occurs. A lung ultrasound examination of a healthy neonate is seen in Supplementary file Video S1.

Furthermore, determination of a LUS-score has been proven to help the clinician make decisions based on the lung ultrasound findings. First described by Brat et al. in neonates, the score was later modified in several ways, but the principle remains the same: For each scanned area, artefacts or even patterns are classified or graded, usually from 0-3. The score of each area is added together, resulting in a score that basically inversely reflects lung aeration [3,16].

Our goal is to provide recommendations about the use of NPLUS especially for the critically ill neonate using the ABCDE algorithm. 

## 2. A—Airway

Establishing and maintaining an open airway is essential to achieve postnatal transition and spontaneous breathing, or for further resuscitative actions to be effective [17]. Several methods, including head tilt, jaw thrust, suctioning of secretions, supraglottic devices and tracheal intubation are used to ensure open airways [18]. To assess the treatment response to each method, vital sign monitoring, clinical examination and point of care ultrasound can be used.

Intuitively, point of care ultrasound may be considered to be slower than clinical examination, but studies in adults have shown the contrary. In a prospective randomized study in 106 adult patients undergoing trauma resuscitation, ultrasound was faster in identifying esophageal intubation and confirmation of tracheal intubation compared to five-point auscultation and capnography [19].

In our experience, bilateral ventilation can be assessed within 15 s by verifying lung sliding. However, studies are lacking to provide evidence for a recommendation regarding the use of ultrasound to assess an open airway or tube placement during neonatal resuscitation.

### 2.1. Continuous Positive Airway Pressure

In spontaneously breathing preterm infants, it is recommended to consider continuous positive airway pressure (CPAP) as the initial method of ventilatory support after delivery—using either mask or nasal prongs [17]. NPLUS can be used for the diagnosis of conditions treated with CPAP, such as transient tachypnea of the newborn or respiratory distress syndrome [20]. 

### 2.2. Assisted Ventilation

In adult patients, several studies have shown that scanning for lung sliding using ultrasound is superior to clinical assessment in detecting effective ventilation [21,22]. For this purpose, the major sign in ultrasound is bilateral lung sliding [23]. Effective ventilation—regardless of the device—always results in visible lung sliding in the ventilated lungs. Furthermore, it allows the person performing manual ventilation to have visual feedback.

In experienced hands, confirmation of ventilation by ultrasound is faster compared to auscultation [19]. In neonates, lung sliding presents identical as in paediatric or adult patients, and due to the presence of B-Lines, recognition of lung sliding may be even easier in this patient cohort. However, evidence regarding the feasibility of assessment of effective ventilation using NPLUS is missing for neonates.

In adults, ultrasound assessment of the anterior neck soft tissues by measuring the minimum distance from the hyoid bone to skin surface showed promising results in predicting difficult mask ventilation and difficult laryngoscopy [24]. This correlation still needs to be examined in neonates, and findings may help identifying patients, in whom a difficult mask ventilation must be anticipated.

### 2.3. Assessment of Laryngeal Anatomy

Ultrasound allows for direct visualization of the laryngeal anatomy, vocal cords and arytenoid cartilages, and due to the dynamic character of an ultrasound exam, it can be used for assessment of glottic opening and vocal cord movement. 

Ultrasound can play an important role in the diagnosis of vocal cord palsy and laryngomalacia without exposing the child to the risks of an invasive endoscopy [25,26].

In a recently published article, Oulego-Erroz et al. used ultrasound to diagnose laryngomalacia in an infant with stridor who required respiratory support. The authors successfully captured the collapse of arytenoids and narrowing of the glottic opening when the infant was agitated. The finding was confirmed by endoscopy [27].

### 2.4. Laryngeal Mask

A laryngeal mask can be used in infants above 34 weeks gestation when problems with establishing effective ventilation with a facemask occur, intubation is not possible, or as an alternative to tracheal intubation [17]. Correct positioning of a laryngeal mask is usually assessed by capnography, appropriate chest excursion, and the absence of an audible leak [28]. In 2015, Kim et al. demonstrated that it is possible to correctly identify rotated laryngeal masks in paediatric patients by assessing the symmetry of arytenoid elevations in transversal view using ultrasound. Using the same method, Song et al. were able to confirm laryngeal mask placement by ultrasound to be quick, non-invasive and reliable in adults [29]. Although no study assessed ultrasound for the detection of the correct position of laryngeal masks in neonates, the signs are the same as in paediatric patients. Evidence for a recommendation regarding the laryngeal mask placement during resuscitation in neonates is lacking.

### 2.5. Tracheal Intubation

In adult patients, ultrasound was shown to be useful in airway management including the identification of vocal cord palsy, predicting the optimal size of endotracheal, double lumen tubes including tracheostomy-tubes, evaluating the position of breathing tubes in trachea, main-stem bronchus, or esophagus, in pre-anesthetic airway evaluation and percutaneous cricothyroidotomy [30,31]. Some of these applications have already been studied in paediatric patients and neonates, but data is limited, especially in neonates.

Chest X-ray is considered gold standard for determination of endotracheal tube location and is recommended by the NLS Guidelines [17]. However, it contributes to cumulative radiation exposure, is often time-consuming and requires manipulation of the patient [32]. Verification of endotracheal tube position was shown to be possible using ultrasound in neonates back in 1986 by Slovis and Poland, who were able to directly visualize the tip of the tube in 16 infants. They reported that a distance of less than 1 cm between the tip of the tube and the aortic arch to be too low in the chest. This method was later shown to have 94% (95% CI: 85–98%) concordance with a tube positioned below the third thoracic vertebra in chest-X-ray in 56 cases in 29 neonates with birthweights between 370–3750 g [33]. The most common technique to assess correct endotracheal tube placement in neonates is measuring the distance between the tip of the tube and the superior aspect of the right pulmonary artery, because the carina cannot be reliably identified, and the superior aspect of the right pulmonary artery is approximately at the level of the bottom of the carina on midsagittal view (see Supplementary file Video S2) [32,34,35]. It is important to note that the tip-to-carina distance in chest-X-ray does not account for the angulation of the trachea to horizontal plane of 20° in supine position [34]. This assessment takes between 5 and 19 min and has significant agreement with chest-X-ray in neonates with bodyweights between 560–4935 g (95% CI: 0.92–0.98, *n* = 40), between 485–3345 g (*r*^2^ = 0.68, *n* = 30), and with mean bodyweight of 2037 g (*r*^2^ = 0.61, 95% CI: 0.26–0.79, *n* = 40) [32,34,35,36]. Overall, ultrasound interpretation of the endotracheal tube position in neonates correlates with the radiography position in 73–100% of cases with over 90% concordance with chest-X-ray in the four largest studies performed [37].

While direct visualization of the tip of the tube is accurate, in critical situations time is essential and assessment of bilateral lung sliding may be sufficient for assessment of the placement of the endotracheal tube, as shown by Fajardo-Escolar et al. in a preterm neonate with esophageal atresia. Even during resuscitation of a neonate, esophageal intubation can be detected using transversal view of the trachea and neck [37,38,39]. In our experience, assessment for bilateral lung sliding can be performed within one minute.

### 2.6. Ultrasound Guided Tracheal Intubation

In 2012 Fiadjoe et al. used real-time ultrasound of the vocal cords while performing a jaw thrust and inserting a hockey-stick-shaped styletted tube in the midline of the patients pharynx under direct vision until it was visible in ultrasound to successfully intubate a 14-month old child and called this technique ultrasound guided tracheal intubation [40]. This technique was later reported by Moustafa et al. to be 99%, 132/133 (72%, 96/133 on the first attempt) effective needing 57 s in adult patients [41]. This high efficacy was later confirmed by Ma et al., who found a 93.3% (28/30) success rate [42].

All studies used a hypoechoic shadowing and widening of the vocal cords in transversal view as proof of endotracheal intubation [41,42,43]. 

Although this technique was only studied in case reports in neonates, it is a promising alternative in patients with difficult airways, especially when resources are limited.

### 2.7. Airway Obstruction

Airway obstruction can be caused by inappropriate positioning with decreased airway tone and/or laryngeal adduction, or due to mucus, vernix, meconium, or blood clots [17]. By using NPLUS, not only effective ventilation by bilateral lung sliding can be recognized, but this method also provides the clinician with additional information regarding the cause for deterioration and produces an objective and reproducible image.

### 2.8. DOPES

The PLS Guidelines state that sudden rapid deterioration of a child with ventilatory support (via mask or tracheal tube) is a time-critical event that demands immediate action and recommends “DOPES” as a mnemonic for displacement of the device, obstruction of the airway, pneumothorax, equipment failure or stomach for abdominal compartment [7]. In assessing for “DOPES”, ultrasound can be used for the first four letters simultaneously by searching for bilateral lung sliding. If present, the lung is bilaterally ventilated, excluding displacement of the device or obstruction of the airway and pneumothorax and most cases of equipment failure. 

## 3. B—Breathing

The focus of LUS concerns the B-part in the ABCDE algorithm. Based on different clinical situations, we try to describe the possibilities of NPLUS in this category.

### 3.1. NPLUS in the Delivery Room 

The choice of appropriate measures for the management of the initial stabilization process of preterm and term neonates is an important aspect in neonatology. Changes in aeration and subsequent hemodynamic changes happen quickly and must be evaluated consistently. Ventilation strategy, and evaluation of adequate lung aeration are key aspects in which ultrasound can be useful.

One of the most crucial steps in interpreting NPLUS is the appearance of the pleural line and lung sliding, as mentioned above. Recognition of lung sliding can be used to determine ventilation, rule out pneumothorax or identify the lung point as indicated in the Bedside Lung Ultrasound in Emergency (BLUE) Protocol or Sonographic Assessment of liFe-threatening Emergencies (SAFE) Algorithm [3,9].

Several studies analyzed the usefulness and reliability of NPLUS in the delivery room. Blank et al. used NPLUS to depict and analyze the initiation of breathing in term and late preterm infants. The authors captured one of the first four breaths in 35 infants and even the first breath in 28 infants. Using a modified, semiquantitative score with several different types of patterns, including “type 0” which occurs prior to establishment of the pleural line. The authors concluded that after the first four breaths, all infants had a visible pleural line, and that fluid clearance happens quickly with establishment of inspiratory efforts. NPLUS can be used to confirm initial penetration of air into the lungs and may support the decision to use different ventilation strategies. A white lung in the delivery room has been predictive for further need of respiratory support and surfactant therapy in this study [44]. In a more recent study, Blank et al. analyzed LUS videos of term and late preterm infants during the first day to describe the transition process, confirming that fluid clearance can be seen during the first few minutes of life with complete clearance after 4 h of life [45]. In another study, NPLUS was performed in the delivery room in 52 very—or extremely preterm infants. Findings included an excellent specificity for surfactant application, and even after 5–10 min, NPLUS outperformed the widely used FiO_2_threshold for prediction of surfactant therapy [46]. Raimondi et al. described fluid clearance in neonates > 33 weeks using 3 types of patterns (white lung, prevalence of B-Lines, prevalence of A-Lines) and presented the shift from white lung to prevalence of A-Lines. White lung pattern performed as a predictor for NICU admission with a sensitivity of 77.1% and specificity of 100% [47].

In our experience, it is challenging to use NPLUS to visualize the first few breaths routinely due to practical reasons (positioning of the ultrasound machine, timing of examination, lack of new information due to clinical features). However, we use NPLUS for diagnostic purposes when signs of respiratory insufficiency, and/or tachypnoea occur, or in situations without sufficient ventilation. 

### 3.2. Infants with Respiratory Distress: Respiratory Distress Syndrome vs. Transient Tachypnea of the Newborn

Respiratory distress is one of the most common reasons for admission to the NICU, therefore it is of great interest to differentiate between respiratory distress syndrome (RDS) with need for surfactant and transient tachypnea of the newborn (TTN) with delayed fluid clearance [48,49]. Several studies address the role of NPLUS in this situation. One of the first observations in this matter was described by Copetti and is called the “double lung point” which is seen in TTN [50]. This sign describes the boundary between coalescent B-Lines or white lung and aerated lung with A-Lines. However, more recent studies have shown that specificity and sensitivity of this sign was lower than described first. When respiratory distress occurs due to TTN, NPLUS would typically show a white lung with a great variability in different lung fields, disappearance of A-Lines, double lung point, irregular pleural line and pleural effusion in 20% of cases, while the occurrence of consolidations would point to RDS, MAS or pneumonia [51,52,53]. Ultrasound is as accurate as chest-X-ray in detecting TTN [54].

However, when RDS is present, sonographic findings differ from TTN: In RDS, typical findings are bilateral white lungs without spared areas or a boundary to a better aerated lung, consolidations, and a thickened, irregular pleural line [3]. One has to keep in mind that TTN and RDS are not always exclusively present and there might be mixed forms, which need a continuous clinical evaluation. See Supplementary file Video S3 for an infant with RDS, and Supplementary file Video S4 for an infant presenting with TTN.

### 3.3. Extremely Preterm Infant After Delivery—LUS and the Need for Surfactant and Ventilation

There is excellent evidence for the use of NPLUS to recognize the need for ventilation and surfactant replacement in preterm infants as described below.

In 2008, Copetti et al. studied NPLUS in infants and emphasized its value in detecting RDS, proposing that LUS may be helpful for guiding surfactant administration [10]. Two years later, the authors demonstrated that surfactant therapy in very preterm infants with a white lung in NPLUS did not improve interstitial fluid clearance [55]. Raimondi et al. studied 54 newborns (mean gestational age 32 weeks) who were admitted to the NICU with nasal CPAP and showed that a sonographic white lung predicted intubation within 24 h with a sensitivity of 88.9% and sensitivity of 100% [56].

De Martino et al. included 133 extremely preterm infants in a prospective trial and were able to show that NPLUS can be used to predict the need for surfactant. The authors demonstrated that it is possible to guide surfactant therapy using a LUS Score with a sensitivity of 82% and specificity of 92%, and they also provided data for the prediction of a second dose of surfactant [57]. In summary, the authors concluded that NPLUS guided surfactant administration is a reasonable alternative to FiO_2_criteria. This hypothesis was confirmed in the ULTRASURF study by Rodriguez-Fanjul et al., in which 56 infants were randomized in two groups, receiving ultrasound-guided or FiO_2_-guided surfactant treatment, respectively. They concluded that the ultrasound-guided group received surfactant earlier and thus was exposed to less supplemental oxygen than the FiO_2_-guided group [58].

The European consensus guidelines on the management of respiratory distress syndrome, updated in 2019, recommend surfactant therapy in infants requiring 6 cmH_2_0 nasal CPAP and an FiO_2_ above 0.3 [59]. There is evidence that the use of the proposed LUS score has an excellent positive predictive value for the need of surfactant treatment and may lead to earlier administration of surfactant in children who need this treatment. Although NPLUS is a comparatively easy ultrasound examination, it still is an observer-dependent method, opposed to the FiO_2_criteria, and one has to be aware of the possibility of differences in management due to inexperienced observers and/or incorrect exam results.

### 3.4. Meconium Aspiration

In infants who require intensive care due to meconium aspiration, NPLUS findings are characterized by atelectases, irregular pleural line, and spared areas, since meconium is distributed unevenly in the lungs and unaffected areas are ventilated properly or are even overdistended. The typical LUS pattern in MAS combines a broad range of signs as seen on Supplementary file Video S5 [3,15]. 

### 3.5. Pneumothorax

Detection and exclusion of pneumothorax are one of the most practical diagnostic features of NPLUS with extensive data regarding sensitivity and specificity [14,60,61]. NPLUS is as accurate as chest-X-ray in the detection of pneumothorax in neonates [62]. Exclusion of pneumothorax is achieved by observation of lung sliding and/or the occurrence of B-Lines. Detection of PTX is possible by absence of lung sliding, detection of the lung point, which can be seen as the boundary between the seashore-sign and stratosphere-sign (also called barcode-sign) in M-mode, as well as mirrored ribs, as seen in Supplementary file Video S6 and Image S1 [63]. In summary, detection of PTX using NPLUS showed excellent specificity and sensitivity.

A further value of NPLUS in pneumothorax commonly used within our units is the possibility of quick and repeated follow-up examinations. Once the lung point is found, the skin can be marked at that exact point and can be re-assessed later with information regarding changes of the size of the pneumothorax (the more lateral the point can be found in supine position, the greater is the dimension of the pneumothorax).

### 3.6. NPLUS in Infants with Bronchopulmonary Dysplasia 

While respiratory distress is the most common reason for admission to the NICU, bronchopulmonary dysplasia (BPD) is the most common chronic disease following prematurity with increasing BPD rates in high-income countries [64].

Infants with BPD can deteriorate quickly due to structural abnormalities, reduced lung volume, high airway resistance and comorbidities of prematurity, resulting in emergency situations where respiratory status has to be evaluated quickly [65]. Increased ventilatory support may lead to pneumothorax, overdistension, bullae and other complications of invasive and non-invasive ventilation. 

NPLUS can be helpful in the assessment of infants with BPD with acute respiratory deterioration. BPD is associated with a higher incidence of RSV infection rates, higher mortality and longer hospital stay [66]. In this context, NPLUS is a reliable tool for diagnosis and follow-up in acute bronchiolitis cases [67]. Findings are primarily consolidations in the posterior chest and pleural effusion, and there is a good correlation between oxygen support and NPLUS findings [68].

On the other hand, regarding the chronic aspect of the disease, there is solid data about the value of NPLUS for the prediction of BPD. Some recent prospective trials analyzed NPLUS scores of infants, who later developed BPD and found good predictive accuracy [69,70]. See Supplementary file Video S7 for an infant with BPD.

### 3.7. Pneumonia

NPLUS shows high diagnostic accuracy for the diagnosis of pneumonia in term and preterm neonates and in the paediatric population [71,72]. Typical signs are consolidations, which are usually larger than in bronchiolitis or RDS, air bronchograms, and pleural effusion [73,74]. 

### 3.8. Diaphragm Movement

Respiratory distress can be caused by abnormal diaphragmatic motion due to diaphragmatic paralysis. M-Mode can be used to assess diaphragmatic movement, achieved by scanning in the subxiphoid plane [75]. A prospective study of 400 healthy children described reference values for diaphragmatic excursion, with values of 6.4 and 6.6 mm excursion for the right and left hemidiaphragm, respectively [76]. Paradox or no movement can lead to the diagnosis of diaphragmatic paralysis in infants with signs of respiratory distress.

### 3.9. Congenital Malformations

Congenital pulmonary airway malformation and congenital diaphragmatic hernia (CDH) are rare malformations and frequently diagnosed antenatally. NPLUS can be used to confirm antenatally diagnosed, or to detect previously unknown cystic lung lesions. Presence of parenchymatous organs or bowel movement inside the thorax leads to the diagnosis of CDH (see Supplementary file Video S8) [77,78].

## 4. C—Circulation

Management of a critically ill infant with circulatory failure, in accordance with the ABCDE approach, should always include proper management of airway, oxygenation and ventilation. After steps A and B, pulse rate, pulse volume, peripheral and end-organ perfusion (capillary refill time, urinary output, level of consciousness), blood pressure and preload need to be evaluated, mainly by clinical examination and echocardiography [79]. In the management of circulatory deterioration, one has to consider etiology, pathophysiology and comorbidities. The transition from a compensated state to decompensation may occur rapidly and be unpredictable [7].

As mentioned above, the current paediatric CPR guidelines recommend performing point-of-care ultrasound by competent providers to identify reversible causes of cardiac arrest (tension pneumothorax, tamponade, hypovolemia). However, its application should not increase hands-off time or impact quality of CPR. Therefore, it is crucial for the team to plan and anticipate the best possible time for sonographic imaging [7,80].

Current evidence supports use of ultrasound for various diagnostic and procedural applications, including diagnosis and monitoring of common pulmonary diseases, hemodynamic instability, patent ductus arteriosus and persistent pulmonary hypertension of the newborn [81].

### NPLUS in Neonates with Congenital Heart Disease and after Cardiac Surgery

Pulmonary overflow is one of the most common complications in patients with congenital heart disease with an incidence of approximately 48–60% [82]. NPLUS may be helpful in newborns with congenital heart disease to assess pulmonary overflow during the first days of life. Neonates with congenital heart disease who tend to develop pulmonary overflow had a higher LUS score at 72 h of life with a good correlation with echocardiography findings and with a better sensitivity and negative predictive value than chest X-ray [83].

Cardiorespiratory complications are common after cardiac surgery. Cardiopulmonary bypass during cardiac surgery generates a systemic capillary leak syndrome with pulmonary edema. NPLUS is a useful tool in monitoring these patients. The use of LUS reduces the exposure to ionizing radiation [8,84].

Diaphragmatic paralysis after cardiac surgery is a major differential diagnosis to consider in case of respiratory insufficiency in NICU patients. Assessment of the diaphragm with NPLUS is described above.

Furthermore, after cardiac surgery, a common reason for clinical deterioration may be pleural effusion or pneumothorax while a chest drainage is still in place. In this situation, NPLUS is helpful for diagnosis and treatment, and therapeutic interventions can be evaluated immediately.

## 5. Future Perspectives and Limitations

As mentioned earlier, the application of bedside LUS in the intensive care setting has gained enormous momentum during the last decade, additionally triggered by the SARS-CoV-2 pandemic. Despite the popularity of bedside ultrasound, more evidence, especially regarding the usefulness NPLUS and associated benefits for term and preterm infants is required, in order to generate recommendations and/or guidelines for the application of NPLUS in neonates. A glance into the literature about paediatric and adult intensive care medicine helps us understand possible applications and future directions when it comes to the further implementation of “visual medicine” in the emergency or intensive care setting. As discussed in the airway-section of this review, there is only limited data on sonography-based assessment of the airway, such as before and after intubation. Lung sliding is a basic, both visual and objective way of confirming effective ventilation, and more studies are needed to assess the benefits or disadvantages of this method in neonatal emergency situations. 

Proper training in ultrasound is a key challenge when it comes to implementation of this method. In the hands of an experienced clinician, a NPLUS exam takes only about two minutes in order to gain important information like lung sliding or pleural effusion. The greatest limitation of ultrasound is the interobserver variability, as it is an observer dependent examination, and especially in the emergency setting, misinterpretation of NPLUS can lead to delayed or even wrong decisions (e.g., placement of a drainage, administration of surfactant, intubation). On the other hand, NPLUS is a comparatively simple diagnostic technique with a steep learning curve and consists mainly of pattern-recognition. Therefore, we consider a theoretical course of 30–60 min and 25 NPLUS examinations under supervision as sufficient for a clinician to count as experienced in NPLUS, based on suggestions by Rouby et al. and Benchouli et al. [85,86].

The development and availability of handheld and/or wireless ultrasound probes gives the neonatologist the opportunity to be more flexible and even quicker when a question about the respiratory status of an infant occurs and sonographic assessment is desired [87]. As mentioned above, the regular use of NPLUS has the potential to reduce the infant’s exposure to radiation significantly [88].

Since we started performing NPLUS as a routine diagnostic tool in our units, we naturally used it increasingly also in emergency settings. It helped us recognize or rule out pneumothorax, visualize ventilation or atelectases, effusions, or consolidations in less than one minute. More experienced sonographers use NPLUS for assessment of tracheal tube placement, more sophisticated questions such as diagnosis of congenital malformations, diaphragmatic movement, and pneumomediastinum as well as assessment of laryngeal anatomy. Furthermore, due to its dynamic properties, NPLUS is an excellent tool for frequent follow-up exams, and visualization of respiratory conditions.

## Supplementary Materials

The following are available online at https://www.mdpi.com/article/10.3390/children8080628/s1. Video S1: Lung ultrasound in anterior view of a healthy neonate. Video S2: Midsagittal view of a neonate at 26 4/7 weeks gestation with an endotracheal tube (ETT) in correct position as confirmed by chest-X-ray. Below the non-ossified sternum, the thymus and the aorta, the hyperechogenic rough line connecting the tip of the endotracheal tube (ETT) with the carina located below the right pulmonary artery (RPA) is the trachea. In practice, the endotracheal tube (ETT) can be identified by the black shadow below or by slightly moving the tube while performing the ultrasound scan. Video S3: Preterm infant with RDS: evenly distributed, confluent B-Lines and thickened pleural line Video S4: Preterm infant with TTN: Unevenly distributed B-Lines, especially in inferior regions, sudden appearance of A-Lines in superior regions, therefore presence of double lung point. Video S5: Video of an infant with MAS. Unevenly distributed areas with irregular pleural line, consolidations, B-Lines, and pleural effusion Video S6: Lung point in M-Mode of a neonate with pneumothorax. The lung point is a highly specific sign for pneumothorax and is found at the margin of pneumothorax and expanded lung. Video S7: Extremely preterm infant at 2 weeks after birth with early signs of bronchopulmonary dysplasia. Typical findings are a thickened pleural line, confluent B-Lines and consolidations in the lower, posterior regions Video S8: Preterm infant with prenatally diagnosed CDH. In a postnatally performed NPLUS exam, bowel movements can be seen to confirm the diagnosis Image S1: Neonate with pneumothorax in B-Mode. Stratosphere sign and mirrored ribs are typical signs of pneumothorax in neonates.

## References

[1] Morton S.U., Brodsky D. (2016). Fetal Physiology and the Transition to Extrauterine Life. Clin. Perinatol..

[2] Sinha S.K., Donn S.M. (2006). Fetal-to-neonatal maladaptation. Semin. Fetal Neonatal Med..

[3] Raimondi F., Yousef N., Migliaro F., Capasso L., De Luca D. (2018). Point-of-care lung ultrasound in neonatology: Classification into descriptive and functional applications. Pediatr. Res..

[4] Whitson M.R., Mayo P.H. (2016). Ultrasonography in the emergency department. Crit. Care.

[5] Bøtker M.T., Jacobsen L., Rudolph S.S., Knudsen L. (2018). The role of point of care ultrasound in prehospital critical care: A systematic review. Scand. J. Trauma Resusc. Emerg. Med..

[6] Singh Y., Tissot C., Fraga M.V., Yousef N., Cortes R.G., Lopez J., Sanchez-de-Toledo J., Brierley J., Colunga J.M., Raffaj D. (2020). International evidence-based guidelines on Point of Care Ultrasound (POCUS) for critically ill neonates and children issued by the POCUS Working Group of the European Society of Paediatric and Neonatal Intensive Care (ESPNIC). Crit. Care.

[7] Van de Voorde P., Turner N.M., Djakow J., de Lucas N., Martinez-Mejias A., Biarent D., Bingham R., Brissaud O., Hoffmann F., Johannesdottir G.B. (2021). European Resuscitation Council Guidelines 2021: Paediatric Life Support. Resuscitation.

[8] Küng E., Ptacek L., Werther T., Reithmayr S., Aichhorn L. (2020). Neonatologie: Lungenultraschall Standard (Version 2.1).

[9] Lichtenstein D.A., Mauriat P. (2012). Lung Ultrasound in the Critically Ill Neonate. Curr. Pediatr. Rev..

[10] Copetti R., Cattarossi L., Macagno F., Violino M., Furlan R. (2008). Lung Ultrasound in Respiratory Distress Syndrome: A Useful Tool for Early Diagnosis. Neonatology.

[11] Stuhlfauth K. (1951). Reflex effects of ultrasonics on the pneumothorax lung. Dtsch. Med. Wochenschr..

[12] Joyner C.R., Miller L.D., Dudrick S.J., Eskin D.J., Bloom P. (1967). Reflected ultrasound in the study of diseases of the chest. Trans. Am. Clin. Climatol. Assoc..

[13] Joyner C.R., Herman R.J., Reid J.M. (1967). Reflected ultrasound in the detection and localization of pleural effusion. JAMA.

[14] Lichtenstein D.A., Menu Y. (1995). A Bedside Ultrasound Sign Ruling Out Pneumothorax in the Critically III. Chest.

[15] Corsini I., Parri N., Gozzini E., Coviello C., Leonardi V., Poggi C., Giacalone M., Bianconi T., Tofani L., Raimondi F. (2019). Lung Ultrasound for the Differential Diagnosis of Respiratory Distress in Neonates. Neonatology.

[16] Brat R., Yousef N., Klifa R., Reynaud S., Aguilera S.S., De Luca D. (2015). Lung Ultrasonography Score to Evaluate Oxygenation and Surfactant Need in Neonates Treated With Continuous Positive Airway Pressure. JAMA Pediatr..

[17] Madar J., Roehr C.C., Ainsworth S., Ersdal H., Morley C., Rüdiger M., Skåre C., Szczapa T., te Pas A., Trevisanuto D. (2021). European Resuscitation Council Guidelines 2021: Newborn resuscitation and support of transition of infants at birth. Resuscitation.

[18] von Ungern-Sternberg B.S., Erb T.O., Frei F.J. (2006). Management der oberen Atemwege beim spontan atmenden Kind: Eine Herausforderung für den Anästhesisten. Anaesthesist.

[19] Mishra P., Bhoi S., Sinha T. (2018). Integration of point-of-care ultrasound during rapid sequence intubation in trauma resuscitation. J. Emergencies Trauma Shock.

[20] Raimondi F., Yousef N., Rodriguez Fanjul J., De Luca D., Corsini I., Shankar-Aguilera S., Dani C., Di Guardo V., Lama S., Mosca F. (2019). A Multicenter Lung Ultrasound Study on Transient Tachypnea of the Neonate. Neonatology.

[21] Álvarez-Díaz N., Amador-García I., Fuentes-Hernández M., Dorta-Guerra R. (2015). Comparación entre la ecografía pulmonar transtorácica y el método clínico para confirmar la posición del tubo de doble luz izquierdo en anestesia torácica. Estudio piloto. Rev. Española Anestesiol. Y Reanim..

[22] Pfeiffer P., Rudolph S.S., Børglum J., Isbye D.L. (2011). Temporal comparison of ultrasound vs. auscultation and capnography in verification of endotracheal tube placement: Lung ultrasound for ET tube verification. Acta Anaesthesiol. Scand..

[23] Marciniak B., Fayoux P., Hébrard A., Krivosic-Horber R., Engelhardt T., Bissonnette B. (2009). Airway Management in Children: Ultrasonography Assessment of Tracheal Intubation in Real Time?. Anesth. Analg..

[24] Alessandri F., Antenucci G., Piervincenzi E., Buonopane C., Bellucci R., Andreoli C., Alunni Fegatelli D., Ranieri M.V., Bilotta F. (2019). Ultrasound as a new tool in the assessment of airway difficulties: An observational study. Eur. J. Anaesthesiol..

[25] Daniel S.J., Bertolizio G., McHugh T. (2020). Airway ultrasound: Point of care in children—The time is now. Pediatr. Anesth..

[26] Friedman E.M. (1997). Role of Ultrasound in the Assessment of Vocal Cord Function in Infants and Children. Ann. Otol. Rhinol. Laryngol..

[27] Oulego-Erroz I., Terroba-Seara S., Alonso-Quintela P., Benavent-Torres R., Castro-Vecino P.D., Martínez-Saez de Jubera J. (2020). Bedside Airway Ultrasound in the Evaluation of Neonatal Stridor. J. Pediatr..

[28] Kim J., Kim J.Y., Kim W.O., Kil H.K. (2015). An Ultrasound Evaluation of Laryngeal Mask Airway Position in Pediatric Patients: An Observational Study. Anesth. Analg..

[29] Song K., Yi J., Liu W., Huang S., Huang Y. (2016). Confirmation of laryngeal mask airway placement by ultrasound examination: A pilot study. J. Clin. Anesth..

[30] You-Ten K.E., Siddiqui N., Teoh W.H., Kristensen M.S. (2018). Point-of-care ultrasound (POCUS) of the upper airway. Can. J. Anesth..

[31] Osman A., Sum K.M. (2016). Role of upper airway ultrasound in airway management. J. Intensiv. Care.

[32] Zaytseva A., Kurepa D., Ahn S., Weinberger B. (2020). Determination of optimal endotracheal tube tip depth from the gum in neonates by X-ray and ultrasound. J. Matern. Fetal Neonatal Med..

[33] Chowdhry R., Dangman B., Pinheiro J.M.B. (2015). The concordance of ultrasound technique versus X-ray to confirm endotracheal tube position in neonates. J. Perinatol..

[34] Dennington D., Vali P., Finer N.N., Kim J.H. (2012). Ultrasound Confirmation of Endotracheal Tube Position in Neonates. Neonatology.

[35] Najib K., Pishva N., Amoozegar H., Pishdad P., Fallahzadeh E. (2016). Ultrasonographic confirmation of endotracheal tube position in neonates. Indian Pediatr..

[36] Sethi A., Nimbalkar A., Patel D., Kungwani A., Nimbalkar S. (2014). Point of care ultrasonography for position of tip of endotracheal tube in neonates. Indian Pediatr..

[37] Boretsky K.R. (2018). Images in Anesthesiology: Point-of-care Ultrasound to Diagnose Esophageal Intubation. Anesthesiology.

[38] Fajardo-Escolar A.P., Bonilla-Ramírez A.J., Winograd Gómez V. (2018). Ultrasound-guided selective intubation in a preterm neonate undergoing type-C esophageal athresia correction. Case report: Colomb. J. Anesthesiol..

[39] Sim S.-S., Sun J.-T., Fan C.-M., Tsai K.-C. (2016). The utility of ultrasonography to confirm proper endotracheal tube placement in neonates. Resuscitation.

[40] Fiadjoe J.E., Stricker P., Gurnaney H., Nishisaki A., Rabinowitz A., Gurwitz A., McCloskey J.J., Ganesh A. (2012). Ultrasound-guided Tracheal Intubation. Anesthesiology.

[41] Moustafa M.A., Arida E.A., Zanaty O.M., El-tamboly S.F. (2017). Endotracheal intubation: Ultrasound-guided versus fiberscope in patients with cervical spine immobilization. J. Anesth..

[42] Ma Y., Wang Y., Shi P., Cao X., Ge S. (2020). Ultrasound-guided versus Shikani optical stylet-aided tracheal intubation: A prospective randomized study. BMC Anesthesiol..

[43] Kundra P., Padala S.R.A.N., Jha A.K. (2021). Ultrasound guided tracheal intubation with a styleted tracheal tube in anticipated difficult airway. J. Clin. Monit. Comput..

[44] Blank D.A., Rogerson S.R., Kamlin C.O.F., Fox L.M., Lorenz L., Kane S.C., Polglase G.R., Hooper S.B., Davis P.G. (2017). Lung ultrasound during the initiation of breathing in healthy term and late preterm infants immediately after birth, a prospective, observational study. Resuscitation.

[45] Blank D.A., Kamlin C.O.F., Rogerson S.R., Fox L.M., Lorenz L., Kane S.C., Polglase G.R., Hooper S.B., Davis P.G. (2018). Lung ultrasound immediately after birth to describe normal neonatal transition: An observational study. Arch. Dis. Child. Fetal Neonatal Ed..

[46] Badurdeen S., Kamlin C.O.F., Rogerson S.R., Kane S.C., Polglase G.R., Hooper S.B., Davis P.G., Blank D.A. (2021). Lung ultrasound during newborn resuscitation predicts the need for surfactant therapy in very- and extremely preterm infants. Resuscitation.

[47] Raimondi F., Migliaro F., Sodano A., Umbaldo A., Romano A., Vallone G., Capasso L. (2012). Can neonatal lung ultrasound monitor fluid clearance and predict the need of respiratory support?. Crit. Care.

[48] Haidari E.S., Lee H.C., Illuzzi J.L., Phibbs C.S., Lin H., Xu X. (2021). Hospital variation in admissions to neonatal intensive care units by diagnosis severity and category. J. Perinatol..

[49] Battersby C., Michaelides S., Upton M., Rennie J.M. (2017). Term admissions to neonatal units in England: A role for transitional care? A retrospective cohort study. BMJ Open.

[50] Copetti R., Cattarossi L. (2007). The ‘Double Lung Point’: An Ultrasound Sign Diagnostic of Transient Tachypnea of the Newborn. Neonatology.

[51] Liu J., Wang Y., Fu W., Yang C.-S., Huang J.-J. (2014). Diagnosis of Neonatal Transient Tachypnea and Its Differentiation From Respiratory Distress Syndrome Using Lung Ultrasound. Medicine.

[52] Liu J., Chen X.-X., Li X.-W., Chen S.-W., Wang Y., Fu W. (2016). Lung Ultrasonography to Diagnose Transient Tachypnea of the Newborn. Chest.

[53] Vergine M., Copetti R., Brusa G., Cattarossi L. (2014). Lung Ultrasound Accuracy in Respiratory Distress Syndrome and Transient Tachypnea of the Newborn. Neonatology.

[54] Volpicelli G., Elbarbary M., Blaivas M., Lichtenstein D.A., Mathis G., Kirkpatrick A.W., Melniker L., Gargani L., Noble V.E., International Liaison Committee on Lung Ultrasound (ILC-LUS) for the International Consensus Conference on Lung Ultrasound (ICC-LUS) (2012). International evidence-based recommendations for point-of-care lung ultrasound. Intensive Care Med..

[55] Cattarossi L., Copetti R., Poskurica B., Miserocchi G. (2010). Surfactant administration for neonatal respiratory distress does not improve lung interstitial fluid clearance: Echographic and experimental evidence. J. Perinat. Med..

[56] Raimondi F., Migliaro F., Sodano A., Ferrara T., Lama S., Vallone G., Capasso L. (2014). Use of Neonatal Chest Ultrasound to Predict Noninvasive Ventilation Failure. Pediatrics.

[57] De Martino L., Yousef N., Ben-Ammar R., Raimondi F., Shankar-Aguilera S., De Luca D. (2018). Lung Ultrasound Score Predicts Surfactant Need in Extremely Preterm Neonates. Pediatrics.

[58] Rodriguez-Fanjul J., Jordan I., Balaguer M., Batista-Muñoz A., Ramon M., Bobillo-Perez S. (2020). Early surfactant replacement guided by lung ultrasound in preterm newborns with RDS: The ULTRASURF randomised controlled trial. Eur. J. Pediatr..

[59] Sweet D.G., Carnielli V., Greisen G., Hallman M., Ozek E., te Pas A., Plavka R., Roehr C.C., Saugstad O.D., Simeoni U. (2019). European Consensus Guidelines on the Management of Respiratory Distress Syndrome—2019 Update. Neonatology.

[60] Alrajab S., Youssef A.M., Akkus N.I., Caldito G. (2013). Pleural ultrasonography versus chest radiography for the diagnosis of pneumothorax: Review of the literature and meta-analysis. Crit. Care.

[61] Cattarossi L., Copetti R., Brusa G., Pintaldi S. (2016). Lung Ultrasound Diagnostic Accuracy in Neonatal Pneumothorax. Can. Respir. J..

[62] Fei Q., Lin Y., Yuan T.-M. (2021). Lung Ultrasound, a Better Choice for Neonatal Pneumothorax: A Systematic Review and Meta-analysis. Ultrasound Med. Biol..

[63] Küng E., Aichhorn L., Berger A., Werther T. (2020). Mirrored Ribs: A Sign for Pneumothorax in Neonates. Pediatr. Crit. Care Med..

[64] Lui K., Lee S.K., Kusuda S., Adams M., Vento M., Reichman B., Darlow B.A., Lehtonen L., Modi N., Norman M. (2019). Trends in Outcomes for Neonates Born Very Preterm and Very Low Birth Weight in 11 High-Income Countries. J. Pediatr..

[65] van Mastrigt E., Kakar E., Ciet P., den Dekker H.T., Joosten K.F., Kalkman P., Swarte R., Kroon A.A., Tiddens H.A.W.M., de Jongste J.C. (2017). Structural and functional ventilatory impairment in infants with severe bronchopulmonary dysplasia. Pediatr. Pulmonol..

[66] Groothuis J.R., Gutierrez K.M., Lauer B.A. (1988). Respiratory syncytial virus infection in children with bronchopulmonary dysplasia. Pediatrics.

[67] Basile V., Di Mauro A., Scalini E., Comes P., Lofù I., Mostert M., Tafuri S., Manzionna M.M. (2015). Lung ultrasound: A useful tool in diagnosis and management of bronchiolitis. BMC Pediatr..

[68] (2019). Di Mauro; Ammirabile; Quercia; Panza; Capozza; Manzionna; Laforgia Acute Bronchiolitis: Is There a Role for Lung Ultrasound?. Diagnostics.

[69] Alonso-Ojembarrena A., Serna-Guerediaga I., Aldecoa-Bilbao V., Gregorio-Hernández R., Alonso-Quintela P., Concheiro-Guisán A., Ramos-Rodríguez A., de las Heras-Martín M., Rodeño-Fernández L., Oulego-Erroz I. (2021). The Predictive Value of Lung Ultrasound Scores in Developing Bronchopulmonary Dysplasia: A Prospective Multicenter Diagnostic Accuracy Study. Chest.

[70] Hoshino Y., Arai J., Miura R., Takeuchi S., Yukitake Y., Kajikawa D., Kamakura T., Horigome H. (2020). Lung Ultrasound for Predicting the Respiratory Outcome in Patients with Bronchopulmonary Dysplasia. Am. J. Perinatol..

[71] Tusor N., De Cunto A., Basma Y., Klein J.L., Meau-Petit V. (2021). Ventilator-associated pneumonia in neonates: The role of point of care lung ultrasound. Eur. J. Pediatr..

[72] Hegazy L.M., Rezk A.R., Sakr H.M., Ahmed A.S. (2020). Comparison of Efficacy of LUS and CXR in the Diagnosis of Children Presenting with Respiratory Distress to Emergency Department. Indian J. Crit. Care Med..

[73] Liu J., Liu F., Liu Y., Wang H.-W., Feng Z.-C. (2014). Lung Ultrasonography for the Diagnosis of Severe Neonatal Pneumonia. Chest.

[74] Chen S.-W., Fu W., Liu J., Wang Y. (2017). Routine application of lung ultrasonography in the neonatal intensive care unit. Medicine.

[75] Epelman M., Navarro O.M., Daneman A., Miller S.F. (2005). M-mode sonography of diaphragmatic motion: Description of technique and experience in 278 pediatric patients. Pediatr. Radiol..

[76] El-Halaby H., Abdel-Hady H., Alsawah G., Abdelrahman A., El-Tahan H. (2016). Sonographic Evaluation of Diaphragmatic Excursion and Thickness in Healthy Infants and Children. J. Ultrasound Med..

[77] Corsini I., Parri N., Coviello C., Leonardi V., Dani C. (2019). Lung ultrasound findings in congenital diaphragmatic hernia. Eur. J. Pediatr..

[78] Yousef N., Mokhtari M., Durand P., Raimondi F., Migliaro F., Letourneau A., Tissières P., De Luca D. (2018). Lung Ultrasound Findings in Congenital Pulmonary Airway Malformation. Am. J. Perinatol..

[79] Singh Y. (2017). Echocardiographic Evaluation of Hemodynamics in Neonates and Children. Front. Pediatr..

[80] Blanco P., Martínez Buendía C. (2017). Point-of-care ultrasound in cardiopulmonary resuscitation: A concise review. J. Ultrasound.

[81] Miller L.E., Stoller J.Z., Fraga M.V. (2020). Point-of-care ultrasound in the neonatal ICU. Curr. Opin. Pediatr..

[82] Rodríguez-Fanjul J., Llop A.S., Balaguer M., Bautista-Rodriguez C., Hernando J.M., Jordan I. (2016). Usefulness of Lung Ultrasound in Neonatal Congenital Heart Disease (LUSNEHDI): Lung Ultrasound to Assess Pulmonary Overflow in Neonatal Congenital Heart Disease. Pediatr. Cardiol..

[83] Picano E., Scali M.C., Ciampi Q., Lichtenstein D. (2018). Lung Ultrasound for the Cardiologist. JACC Cardiovasc. Imaging.

[84] Liu J., Copetti R., Sorantin E., Lovrenski J., Rodriguez-Fanjul J., Kurepa D., Feng X., Cattaross L., Zhang H., Hwang M. (2019). Protocol and Guidelines for Point-of-Care Lung Ultrasound in Diagnosing Neonatal Pulmonary Diseases Based on International Expert Consensus. J. Vis. Exp..

[85] Rouby J.-J., Arbelot C., Gao Y., Zhang M., Lv J., An Y., Chunyao W., Bin D., Valente Barbas C.S., Dexheimer Neto F.L. (2018). Training for Lung Ultrasound Score Measurement in Critically Ill Patients. Am. J. Respir. Crit. Care Med..

[86] Benchoufi M., Bokobza J., Chauvin A., Dion E., Baranne M.-L., Levan F., Gautier M., Cantin D., d’Humières T., Gil-Jardiné C. (2020). Lung Injury in Patients with or Suspected COVID-19: A Comparison between Lung Ultrasound and Chest CT-Scanner Severity Assessments, an Observational Study. medRxiv.

[87] Schmid M., Dodt C. (2018). Lungensonographie in der Notfall- und Intensivmedizin. Med. Klin. Intensivmed. Und Notf..

[88] Escourrou G., De Luca D. (2016). Lung ultrasound decreased radiation exposure in preterm infants in a neonatal intensive care unit. Acta Paediatr..

