# Supplementary figures and images for: The Role of Lung Ultrasound in the Management of the Critically Ill Neonate—A Narrative Review and Practical Guide
Source: Children (Basel). 2021 Jul 24;8(8):628. doi: 10.3390/children8080628 (PMC8391155; doi:10.3390/children8080628)

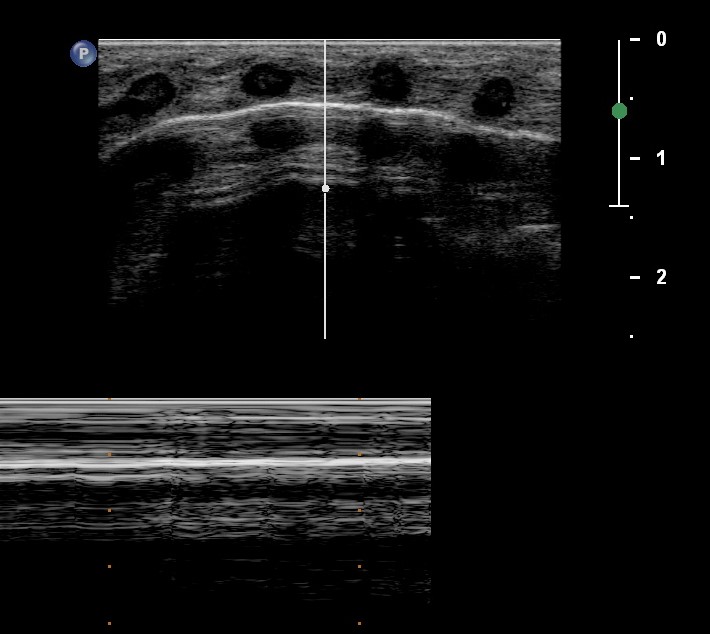

Supplement: Supplementary file 1 [file children-08-00628-s001.zip › ImageS1.jpg]
